# How is Integration Defined and Measured, and what Factors Drive Success in Brazil? An Integrative Review

**DOI:** 10.5334/ijic.7002

**Published:** 2023-10-31

**Authors:** Elaine R. Neiva, Gardenia Abbad, Maria Inês Gandolfo Conceição, Diana Lúcia Moura Pinho, Andreas Xyrichis

**Affiliations:** 1 University of Brasília, BR; 2 Kings College London, UK

**Keywords:** integrated care, review, Brazil, quality of care

## Abstract

**Introduction:**

Integration in health and care can improve quality and outcomes, but it is challenged by expansion of medical knowledge, social pressures on patient needs, and demands to deliver critical information. In Latin American and in other lower and middle-income countries integrated care remains in development. This paper examined the available literature on integrated care to understand how Latin American countries identify and measure integration, and what factors influence success.

**Methods:**

This integrative literature review included systematic searches in Global Health, PubMed, SciELO and BVSPsi databases for articles on integrated care in Spanish, Portuguese, and English in the period from January of 1999 to December 2020. The articles were screened for selection and assessed independently by five reviewers that used the inclusion criteria of papers about integration in health care systems. The sample excluded articles that did not deal with the integration of health care, which addressed issues related to public health campaigns, programs to control endemics and epidemics, reports on the experience of implementing health services, health promotion guidelines, food safety, oral health, and books evaluation.

**Results:**

24 articles were included: qualitative (75%), quantitative (12,5%), and mixed-method research (4%) published between 2000 and 2017. All studies were undertaken in Brazil, and two of them were also conducted in Latin American countries. In 15 articles there was an interchangeable use between concepts of integration of services and integrated care, while nine studies did not define integration. Barriers to integration included absence of shared understanding of knowledge among members of interprofessional teams, lack of clarity on professional roles, missing consensus on a definition and measurement of integrated care, power struggles between professionals, poor institutional support, insufficient team preparation and training and unequal valuation of professions by society.

**Conclusion:**

Several types of integration and factors contributing to the success of implementation of integrated care in various contexts in Brazil were identified. The concept of integration reflected the varied local and regional realities including different health settings and levels of health and care, suggesting a need for further clarifications on its objective and components especially in LMIC contexts.

## Introduction

Internationally, health and care systems face challenges to deliver affordable and high-quality services [1]. The COVID-19 pandemic crisis contributed to an increase in the prevalence of chronic conditions and added to the complexity of treatment pathways, highlighting the importance of coordinating, and integrating health and care services [2,3]. The rapid expansion of medical knowledge exacerbates the difficulties in the dispersal of critical information among professionals co-responsible for patients’ care. Meanwhile, there is rising public pressure for health and care providers to recognize and accommodate individual patients’ medical needs, social environments, and care preferences [4]. With this complex backdrop, integration has emerged as a solution to many of the issues faced by the current fragmentation of health care services. Concepts such as integrated care and integrated services have been suggested as pathways to improve the quality and outcomes of health and care services [5,6,7]. Notwithstanding considerable progress made among high-income countries, integrated care in lower and middle-income countries (LMICs) is still in development. This paper examines the available literature on integrated care with a focus on LMICs and Latin America in particular, with a view to describing the current state of play and proposing future research directions.

## Background

Brazil’s Unified Health System (Sistema Único de Saúde - SUS), widely acknowledged as an example of successful health system reform in Latin America, is regarded as the largest Primary Health Care (PHC) system in the world. Some notable characteristics of SUS are: the universal right to comprehensive health care at all levels of complexity (primary, secondary, and tertiary); decentralization with responsibilities given to the three levels of government: federal, state, and municipal; social participation in formulating and monitoring the implementation of health policies through federal, state, and municipal health councils. Between 2002 and 2013, there was near universal access to essential health services, such as immunizations and antenatal care, with improved population health outcomes, and declines in regional health inequalities [8]. Despite progress, health inequalities persist, mirroring the wealth and income inequalities in the country. These disparities that include access to effective care, financial protection and health outcomes are partly due to structural weaknesses in the health system, such as state government challenges, inadequate funding, and unequal resource allocation [9]. Currently, the SUS is under threat due to a combination of economic recession, political crisis, corruption and mismanagement of public funds, ill-conceived austerity policies and political decisions. Integrated care holds the potential to contain some of these challenges and safeguard SUS’s sustainability.

Health systems across Latin America share some distinguishing features with other LMICs. During the last four decades, Latin American countries have led several initiatives to promote greater integration and cooperation. These initiatives have assumed a rhetoric that recognizes health as relevant to the governance and development functions of nations, as well as the value of South-South cooperation as a tool both for overcoming population? health problems and the strengthening of regional identities within the new world geopolitical order. The implementation of joint price negotiation policies to expand or ensure basic access to medicines is a notable example [8,9,10]. Another shared feature is the support from international organizations to different government initiatives to drive greater cooperation between countries or facilitate the exchange of experiences. Latin America, and Brazil in particular, is characterized by territorial dispersion, with diverse and vulnerable populations present in different and often remote locations. This poses a great challenge to the integration of health and care.

A retrospective review of 40 years of cooperation in health in the subregional contexts of Latin America and the Caribbean [10] revealed a shared identity in the discourse of the different institutional actors who have repeatedly committed themselves through protocols, agreements, and declarations to improve the health conditions of the population of the subregions they represent. Nevertheless, four decades after the creation of the first subregional cooperation initiative in health in Latin America [8,9,10] vis-à-vis the continuity of the same unresolved health problems, demonstrate the fragility of these commitments and of the institutional mechanisms for encouraging compliance.

## Integrated care: some issues

Integrated care is a concept that seeks to unify patient care, services, and organizations, as well as academic work towards creating unified conceptual models. However, the idea of a unified concept is undermined by two main points. First, integrated care is not an empirical phenomenon, but rather comprises a multitude of objects, strategies, and objectives. Efforts towards integrating care are concerned with building unity among a wide range of objects, including patients’ care and experience, multidisciplinary and inter-organizational work, and health and care systems. These efforts include overlapping, interrelated, and sometimes conflicting strategies and experiences, driven by a wide range of purposes. Patients, service providers, and legislators have different ideas and experiences regarding integrated services. Secondly, the conceptual models of integrated care assume the alignment of patients’ and systems’ perspectives, and multiple strategies in a coherent and decontextualized whole. The interchangeable use of concepts such as integrated care or integrated services leads to a need to clarify the object of integration and its core components, so that outcome measures can be developed. A definition of integrated care is needed that differentiates it from the organization of integrated delivery, acknowledging that integrated organizational structures and processes may fail in producing integrated care for the patient.

Integrated care is a broad concept, which can be used to describe a connected set of clinical, organizational, and policy changes aimed at improving service efficiency, patient experience, and outcomes. Integrated care can refer to both the methods that might be used to organize, fund, and deliver health and related services, and the interrelated goals of better outcomes, experiences, and use of resources. Integrated care has been studied in several ways—for example, as an organizational and social process [1], as an indicator of health system effectiveness, and for its effects, such as economic impact. The concept of “integration” is used in a variety of ways and contexts. However, when used in practice there is often ambiguity regarding two issues: a) the object of integration and b) it’s essential components. First, discussions of integrated health care often implicitly conflate delivery systems and delivery processes with their product: patient care. However, organizations, the processes they use to deliver care, and the care patients receive are all conceptually distinct objects to which the term integration can be applied. Integration of organizations and organizational activities may or may not result in integration of the care delivered to patients. Integration was originally used in organizational theory to describe collaborative activities among differentiated units within an organization that enables them nevertheless to achieve “unity of effort” [11].

For the purposes of the current article, we are using integrated care to refers to “an approach for individuals or populations where gaps in care, or poor care coordination, leads to an adverse impact on care experiences and care outcomes” [12]. This approach is particularly relevant for frail older people, those living with long-term chronic and mental health illnesses, and those with medically complex needs or requiring urgent care. However, integrated care should not be viewed solely as a response to managing medical problems, as its principles extend to promoting health and wellbeing more broadly. In practice, integrated care has three distinct dimensions. Firstly, it is a necessary response to overcome fragmentation in care delivery that adversely impacts the ability to coordinate care effectively around people’s needs, resulting in sub-optimal care experiences and outcomes. Secondly, integrated care represents an approach to improve the quality and cost-effectiveness of care by ensuring that services are well-coordinated around people’s needs, making it both people-centered and population-oriented. Finally, the people-centered focus becomes the organizing principle for integrated care as a service innovation, whether it is related to individual patients, their carers/families, or the wider community to which they belong [13,14].

Existing literature reviews on integrated care and integration of health services tend to draw from high-income settings, but a focus on LMICs and Latin America is scarce. The current paper set out to make sense of the literature on integrated care specifically from Latin America, by asking three questions:

1. How does the literature from Latin America define integration? (Q1)
2. Which measures or indicators are used in this literature to infer integration? (Q2)
3. What are the factors that influence the success of integrated care in Latin America? (Q3)

## Method

The article is informed by an integrative literature review approach [8]. A systematic search for literature was carried out in the Global Health, PubMed, SciELO and BVSPsi databases using the following keywords: [integrated care, OR integrated disease management, OR comprehensive health care, OR case management, OR clinical pathways, OR integrated and (health or care or delivery or system), OR teamwork, OR collaboration, OR coordination, OR patient care team] AND [health and care, OR SUS, OR NHS, OR health service, OR health system] AND [lower income, OR middle income, OR ODA eligible, OR developing countries]. The search comprised scientific articles from 1973 until 2020 which were written in Portuguese, Spanish and English. The search has involved only academic and peer reviewed papers, not including material published by governmental or technical reports.

Qualitative, quantitative, and mixed-method studies were all eligible for inclusion. The setting of the chosen studies comprised only LMICs, and studies conducted with health and care stakeholders (professionals, policy makers, service users, patients, family members). The sample excluded articles that did not deal with the integration of health and care, which addressed issues related to public health campaigns, programs to control endemics and epidemics, reports on the experience of implementing health services, health promotion guidelines, food safety, oral health, and books. The final list of selected articles was restricted to Brazil and Latin America.

Search results were independently screened for relevance by at least two reviewers. Excluded articles were discussed through a series of online meetings with final decisions reached by consensus. Two rounds of evaluation were carried out: 1) the titles and abstracts of the articles were analyzed to assess whether they met the eligibility criteria; 2) the entire article was analyzed to verify if it met the purposes of the literature review. In the first phase, the initial 545 articles were reduced to 152, and in the second phase, only 24 articles were kept.

Data were extracted using a predetermined form available in Annex. Key characteristics of the papers, including design, sample, data collection and analysis were extracted as were key results. Five reviewers analyzed all the articles. The synthesis followed an integrative and thematic approach seeking answers to the review questions.

## Results

In total, 24 studies met the eligibility criteria and were included in the review (Table 1). These studies were published between 2000 and 2017. Thirteen (54,2%) were published between 2008 and 2012. Most (n = 22, 92%) were undertaken in various parts of Brazil (Northeast, South, Southeast and Central-West regions). Furthermore, two studies (n = 2, 8%) were conducted in both Brazil and Latin America countries (Argentina, Paraguay, Uruguay, Colombia).

**Table 1:** Characteristics of sample data.


AUTHOR	YEAR	SETTING	RESEARCH DESIGN	INSTRUMENTS/TOOL	PARTICIPANTS	COUNTRY/STATE/REGION

1. Souza MF et al. [28]	2017	Primary care National Program for Primary Care Access and Quality Improvement (PMAC-AB) Team’s work process	Cross-sectional study – use of the Item Response Theory (IRT) (Analyze the quality-External evaluation) Secondary source	Interview Samejima’s Gradual Response Model – level of coordination. PMAQ-AB Questionnaire	17.202 primary care teams	Brazil/Minas Gerais/Southeast

2. Vargas I et al. [8]	2016	Health Systems Focus on three level – coordination across levels	Case study – qualitative, exploratory and descriptive–interpretative study. Multicentric	Semi-structured interviews	health professional (n = 112) administrative (n = 66) professionals of different care levels (??) managers of providers (n = 42) and insurers (n = 14).	Latin America – Brazil and Colombia

3. Pieri FM et al. [29]	2014	Primary care (Family Health Strategy) Chronic disease -leprosy	Cross-sectional study	Questionnaire (adpt) – PHC Assessment Tool (PCATool)	165 patients (leprosy) in the end 119 patients	Brazil/Parana/ South

4. Scherer MD [24]	2013	Training Program Family Care Program in Brazil	Cross-sectional study	Semi-structured interviews, participant observation and focus groups.	16 health professionals	Brazil/Brasilia/Central-West

5. Pinto RM et al. [30]	2012	Primary care (Family Health Program)	Cross-sectional study	In-depth interviews	30 CHWs	Brazil/Rio de Janeiro/Southeast

6. Santos MC, Tesser CD [31]	2012	Primary health and care – Brazilian Unified Health System Integrated and complementary practices – method	Research-action methodology (four stages)	The method involves four stages: 1 – definition of a nucleus responsible for implementation and consolidation thereof; 2 – situational analysis, with definition of the existing competent professionals; 3 – regulation, organization of access and legitimation; and 4 – implementation cycle: local plans, mentoring and ongoing education in health.	112 health teams	Brazil/Santa Catarina/South /São Paulo/Southeast

7. Heimann LS et al. [32]	2011	Primary health and care – (as strategy) stewardship capability, financing, provision, comprehensive and intersectoral	Case study (Multicentric – Argentina, Brazil, Paraguay and Uruguay) Multicentric	Literature review, document analysis and interview	Policy makers, managers, experts, users and professionals (n = 22).	Latin America – Brazil/Argentina, Paraguay/Uruguay

8. Queiroz M, Delamuta L [17]	2011	Mental Health Programs Teams – decision process	Cross-sectional study	Interviews and participant observation	Health professionals (n = 22) Psychologist(n = 4) physician (n = 4), occupational therapists (n = 4), nurses(n = 4), nutritionists (n = 2), 1 social worker (01) nutritionist (n = 01) economist(n = 01) and administrator (n = 01)	Brazil/São Paulo/Southeast

9. Villa et al. [33]	2011	Primary Health Care Health service performance in TB control in Brazil	Cross-sectional study Multicentric	Interviews – Primary Care Assessment Tool adapted for TB	514 patients (TB)	Brazil/São Paulo/Bahia/Paraiba – Southeast and Northeast

10. Costa MC et al. [18]	2010	Network Care – National Program of Integrated and Referential Actions (PAIR) child and adolescent’s sexual violence	Cross-sectional study – Descriptive study	Interviews	Professionals from the Institutions of care (n = 38), Municipal Committee of PAIR (n = 11) and key community informants (n = 78)	Brazil/Bahia/Northeast

11. Kell MC, Shimizu H [27]	2010	Primary care (Family Health Strategy) FH Teams	Qualitative research	Observations, interviews and focus groups	Four complete FH teams (physician, nurse, community health workers, nursing assistants) 32 professionals from the Family Health Strategy	Brazil/Goiás/Central-West

12. Mello AS, Moysés SJ [21]	2010	Pre-hospital assistance (mobile and fixed emergency services) attending accidents and violence against elderly.	Situational analysis (General conditions of infrastructure, planning and support at the pre-hospitalar assistance level elderly – quantitative and qualitative approaches	Interview	4 health managers; 14 health professionals	Brazil/Paraná/ South

13. Moretti AC et al. [34]	2010	Primary Care – (Family Health Strategy) oral health teams Intersectoral actions	Cross-sectional study with quantitative and qualitative methodology	Self-response questionnaire and focus group	94 Family Health Units (oral health team)	Brazil/Paraná/South

14. Araújo MB, Rocha PM [35]	2009	Primary Care (Family Health Strategy) FH Team	Qualitative case study	Semi-structured interviews	Family health units- 06; 25 health professionals 5 physicians; 5 nurses; 2 dentists; 6 community health workers; 6 nursing assistants and 1 dental assistant	Brazil/Rio Grande do Norte/ Northeast

15. Consoli GL et al. [22]	2009	Public health network Mental health and care	Not stated	Structured questionnaires	professional public health care 31 municipalities	Brazil/Rio Grande do Sul/South

16. Giovanella L [36]	2009	Primary Care (Family Health Strategy) FH Team	Four case studies Multicentric	Semi- structured interviews with managers and surveys with health care professionals and registered families.	municipal managers (n =77) professionals from the FH teams with self-administered questionnaires (n = 1,336); registered families with structured questionnaires at home (3,312 families	Brazil/Sergipe/Northeast/Minas Gerais and Espírito Santo/ Southeast//Santa Catarina/ South

17. Franco TB, Merhy EE [37]	2008	Primary care (Home health care) Work process and network	qualitative study	Semi-structured interviews, observation, technical visit and documental analysis	Coordinator of the home care operator, manager of the home care service provider, the coordinator of the teams in the Home Care Program, the workers who make up the teams, beneficiaries or caregivers included in the program.	Brazil/Rio de Janeiro/Southeast

18. Costa-e-Silva V et al. [15]	2007	Primary Care (Family Health Unit) Maternal and child health	Case study used Qualitative approach	Interview semi-structured (regional and central level); Self-completed questionnaire. Workshop	35 health professionals: 23 health family unity – and 6 (specialized centers)	Brazil/Espírito Santo/Southeast

19. Macinko J et al. [25]	2007	Primary care –(Family Health Strategy) Infants mortality rates, Neonatal mortality, Post-neonatal mortality	Pooled cross-sectional ecological analysis Secondary source (follows a quasi-experimental design)	Panel data from Brazilian microregions	557 Brazilian microregions. 3342 observations (6 years 1999-2004)	Brazil (Throughout the country – 27 Brazilian states – regions North, Northeast, Southeast, South and Central-west)

20. de Oliveira EM, Spiri WC [26]	2006	Primary care – (Family Health Strategy) Multiprofessional teamwork	Qualitative approach -phenomenology,	Interviews	Two physicians, two nurses, two nursing assistants, and two community health workers	Brazil/Rio Grande do Sul/ South

21. Baxter YC et al. [16]	2005	Hospital – Integrated home-hospital Model of Integrating Hospital and Home vs Conventional Hospital Model Dehospitalization – nutritional therapy	A retrospective controlled study, paired (age, sex, disease, and surgical procedure) Secondary data	Various sources – Medical record, nutritional attendance form, Finances, Pharmacy Division for Esophageal Surgery	56 documents of patients from Digestive surgery	Brazil/São Paulo/Southeast

22. Gupta N et al. [38]	2005	Primary care – (Home care) Support Domiciliary Therapeutic Assistance program	Quasi experimental design with subjects divided into three groups:	Interview,	56 subjects divided into three groups (G1/DTA = 15 subjects; G2/Previous DTA = 21 and G3/Non DTA = 20)	Brazil/Espírito Santo/Southeast

23. Magnani RJ et al. [20]	2001	Schools and Clinics training courses Adolescent Reproductive Health Program in Bahia – HIV infection	Quasi-experimental control-group panel design	Self-administered surveys, Observation	Students, 300 providers (physicians, nurses, social workers) attended)	Brazil/Bahia/Northeast

24. Pinotti JA et al. [11]	2000	Hospital and Primary Health care (PHC) Women’s health project integration of diagnostic and therapeutic services	Information about the patients obtained in a computerized system	Questionnaire-self organization of an experimental model of primary health and care (Project)	Patients, Nurse assistant, Physician	Brazil/São Paulo/Southeast


Articles summarised based on their key characteristics, e.g. by setting (how many in primary care, acute care, etc.), research design (e.g. how many used case study, cross-sectional, qualitative interviews, etc.), participants (e.g. professionals, patients), country (Brazil, other LMIC), etc.

Fifteen studies were undertaken at the PHC level. Another two were conducted across primary care, secondary and tertiary level of health care [15]. Two were in the setting of schools and training courses [16]. The principal focus within these primary care studies was to address a family health strategy, teamwork process, network and clinical issues [17,18,19]. The most frequent clinical issue was chronic disease (leprosy and tuberculosis); maternal and child health; mental health and care; and women’s health [20,21,22].

Considering design and methods, most studies followed a qualitative (n = 18, 75%) or a cross-sectional approach [22,23,24]. Three studies (12,5%) were based on a quantitative approach as a retrospective controlled study and quasi experimental design [15,25,26]; one study used a mixed method design [18]. Two studies did not state their research approach [16,27].

In 15 studies there was conflation between the concepts of integration and integrated care for the patient, with interchangeable use of these concepts. There was also conflation among mechanisms identified as indicators or as consequences of integration. In nine studies there was no definition of integration.

## Data synthesis

### Q1. How does the literature from Brazil and Latin America identify integration?

Studies referred to integration as a phenomenon between individuals, professionals or activities within work groups. Twenty-one studies mentioned integration involving workgroups and units, mostly within the same organization. Nine of these 21 studies referred to integration considering the work processes within the same organization but involving diverse groups or units. In another eight articles, integration considered groups, units, or spheres of activity across organizations and institutions; here, studies addressed specific health programs that operated in the spheres of municipal and state health departments, or even involved the federal sphere of the Ministry of Health. Some definitions were tautological as they reinforced that integration is composed of mechanisms of integration and coordination between members of a multiprofessional family health team.

In terms of the level of analysis of the object of integration, almost all (20 articles) articles investigated mechanisms of clinical integration between health professionals from the same team. Most (15 articles) studies analyzed coordination processes between groups and units of the same organization. Few (5 articles) studies investigated the integration between institutions/organizations in the provision of care to patients, family members, caregivers, and the community. Among these studies, there are those (only three articles) that analyzed multiple levels of integration (between professionals of the same team, between teams or units of the same organization, and between organizations). One of these studies analyzed processes of integration between organizations in the health system and of articulation with the intersectoral network in primary health care [34]. Another article reported the evaluation of an educational experience of integration between professionals from different sectors (health, education, and social assistance), aimed at promoting the reproductive health of adolescents [26].

Financial, administrative, social, and organizational support processes and activities were mentioned by some authors as features of integration [18,32,33]. Indeed, the integration of the health care system at the macro level of analysis depends on arrangements, alliances, contracts, and networks between organizations [39], as well as agile management mechanisms, material, and physical infrastructure, and virtual and financial support. Functional integration strategies (coordination of non-clinical and support activities that support services) are considered relevant mechanisms to support the integration of care. However, these mechanisms, structures, and non-clinical work proceses have been poorly studied.

An example of a proposal for normative integration defined as the development and maintenance of common references between organizations, professionals, groups, and individuals [39], is the work of Santos and Tesser [29], which proposed a method to implant integrative and comprehensive practices in the Brazilian Unified Health System, based on the principles, values, and mission of SUS. Integration was seen as a consequence of the transition from a disease-focused model to one that has health and quality of life as its guiding axes, and in which communication and integration mechanisms co-exist between care providers. In this transition, changes should occur either at the conceptual or political level, and, essentially, in the field of practices, and in the daily work relations of actors directly involved in this process. Here, integration was noted to comprise integrated patient care in a program delivered by a multiprofessional team.

The articles revealed integration as a multifaceted concept, associated with the interaction between people, groups and units. There is also a shared meaning between integration and integrated service to the patient. Many studies assumed that both phenomena are inextricably linked, with one necessarily leading to the other.

### Q2. Which measures or indicators are used in this literature to infer integration?

The studies mentioned many different objectives of integration, concerning the program’s actions, activities, interaction or connections between professionals, linkages between groups, units, or organizations. Alongside this, the studies also list positive aspects arising from integration in health services. Integration can foster quality care to the patient, as well as other results both at the level of the patient, the professionals, the workgroup and the organization; for example: better patient care; patients’ satisfaction with teamwork in the family health and care program; improvement in services outcomes measures (e.g., mortality); better care received by families; addressing the health needs of the population. Some studies (two studies) have addressed measures to assess the effects of care processes on the reactions of patients, family members and/or caregivers, professionals, or managers [15,33]. Measures of care impacts on patients’ behavior were discussed by some (two authors) authors [8,33].

The participation of users and professionals in the coordination of different care interventions was seen as one indicator to infer integration. The level of coordination was indicated by the existence of a communication channel (telephone/internet), number of institutional communication flows (case discussion, technical meetings with specialist), telehealth usage and participation, electronic medical record, reference/counter-reference file, and electronic communication) [40]. Three types of care coordination were distinguished: information coordination, or transfer and use of patient clinical information needed to coordinate activities between providers; clinical management coordination, or provision of health and care in a sequential and complementary way; and administrative coordination, or coordination of patient’s access to the continuum of health services according to their needs.

### Q3. What are the factors that influence integrated health care in Latin America?

The 25 papers touched on several aspects of integration, with some notable trends identified concerning barriers and facilitators to success. For example, in a study with community health agents [30] knowledge and skills in psychiatric procedures were not regarded as facilitators of integration; rather, it was the shared understanding of practice and ethics that facilitated agreement regarding care planning. Barriers in this case were lack of accountability, commitment, and awareness of the integrated approach by some managers.

The barriers brought up by the articles can be grouped as follows: lack of integration, for example, in primary and secondary care [20]; lack of protocols with preventive approach [20,33]; need for continuous medical supervision [16]; need of constant team conduct negotiation [28]; lack of consensus on the definition [28,40]; lack of clarity about the forms of intervention and measurement of care coordination [40]; hegemonic place conferred to physicians in the professional hierarchy [16,27,28]; deficiencies in case records (33); lack of buy-in to care proposals and poor support institutional framework [27]; impact of regional disparities and social inequality of Brazil on care delivery [30]; unprepared or shortage of appropriately trained health professional to deal with the complexity of health and care problems [28]; deficiencies on health professional courses [28]; insufficient training and low professional experience [28]; low repertoire in interdisciplinary health and care work [28]; incoherence between the theoretical work model and daily work services.

Some studies highlighted that hierarchies and medical dominance are an important barrier to integration [27,28]. A study concluded that the main difficulties encountered in this interaction were related to labor relations resulting from hierarchical powers [27]. Similarly, one of the articles reported that there was an attempt to standardize the conduct of professionals, towards greater integration of work, but factors such as medical guidance in health and care caused tensions and required continuous negotiation around new models of care [28]. The need for continuous medical supervision was also reported, considering that, as one of the studies suggested, even when a nurse assistant trained to perform some specific technical functions, it was difficult for them to make a global analysis of the clinical case and guide the patient accordingly [16].

With integration being applied at different levels [18,34], papers also referred to the importance of intersectoral actions. These refer to initiatives that rely on the interaction of two or more entities belonging to different social or economic sectors within the same territory. Here, reorganization of health services to support interprofessional working was noted, such as through establishing communication protocols among different professions. Team working was a recurring factor noted as an important facilitator to integrated care. Examples of team working initiatives included developing jargon-free communication among professionals and developing processes for professionals to engage with each other in a given place and time. Setting clear expectations and promoting shared understanding of team practice was also noted to help shape accepted norms regarding professional conduct (Table 2).

**Table 2:** Summary of main results.


TOPIC	MAIN RESULTS

Concept of Integration	Studies referred to integration as a phenomenon between individuals, professionals or activities within work groups. Some definitions are tautological as they reinforce that integration is composed of mechanisms of integration and coordination between members of a multiprofessional family health team. There is also a shared comprehension between integration and integrated service to the patient. Many studies assumed that both phenomena are inextricably linked, with one necessarily leading to the other. Almost all articles investigated mechanisms of clinical integration between health professionals from the same team. Most studies analyzed coordination processes between groups and units of the same organization. Few studies investigated the integration between institutions/organizations in the provision of care to patients, family members, caregivers, and the community

Measures or indicators	The participation of users and professionals in the coordination of different care interventions was seen as one indicator to infer integration. Measures to assess the outcomes and benefits of care processes on the reactions of patients, family members and/or caregivers, professionals, or manager. Measures of care impacts on patients’ behavior were discussed by some authors [23] Some indicators as the existence of a communication channel (telephone/internet), number of institutional communication flows (case discussion, technical meetings with specialist), telehealth usage and participation, electronic medical record, reference/counter-reference file and electronic communication).

Factors that influence integrated health care	The barriers brought up by the articles can be grouped as follows: lack of integration, for example, in primary and secondary care; lack of protocols with preventive approach; need for a continuous medical supervision; need of constant team conduct negotiation; lack of a consensual definition; lack of clarity about the forms of intervention and measurement of care coordination; hegemonic place conferred to physicians in the professional hierarchy, deficiencies in case records; unsatisfactory adhesion and poor support institutional framework; impact of regional disparities and social inequality of Brazil on care delivery, unprepared health professional to deal with the complexity of health and care problems; deficiencies on health professional courses; insufficient training and low professional experience; low repertoire in interdisciplinary health and care work; incoherence between the theoretical work model and daily work services.


Facilitators to health care integration identified in the papers were around: establishment of protocols or clinical guidelines [17,20]; creation of ties of commitment to co-responsibility between professionals and the population [20]; good ability to provide/ share information [40]; frequent contact among professionals [40]; use of tools such as telehealth as a second formative opinion, telediagnostic or teleconsulting; delegation of duties to different members of the health team [16]; and attitudes of commitment and critical thinking [28].

In one notable paper, which evaluated Network Care for victims of violence in the period of 2003–2006 [32], results indicated that open, fluid, and clear communication, joint activities, and clear links among different care groups were shown to foster integrated care. Here, the authors also identified financial support, awareness raising campaigns and coordination as important enablers. A key aspect noted to promote integration concerned patient empowerment to be involved and contribute to decisions regarding their care. This could be enhanced through greater collaboration among health professionals involving an interprofessional approach to health and care.

An article on leprosy patients suggested that the establishment of protocols or clinical guidelines can trigger a favorable ambience for bonding, free from discrimination, prejudice and stigma [20]. Similarly, another study pointed out that establishment of bonds and creation of ties of commitment to co-responsibility between professionals and population are essential items [17]. Moreover, the ability to provide information and frequency of contact among professionals are principal elements for a comprehensive, continuous, and high-quality care [40].

Interestingly, among the teams with the best levels of coordination, a large part participated and used telehealth as a second formative opinion, telediagnostic or teleconsulting [40]. One study showed that the key to integrating health and care services for women, with epidemiological interventions to achieve a “comprehensive approach” in PHC, was the delegation of duties to different members of the health team. With such positive results, they highlighted the conviction that delegation of appropriate duties to various competent members of the health team helps achieve quality care, integration and universality [16].

Finally, a study arrived at the conclusion that attitudes of commitment to the care of people, even in the face of adversities, appeared to enhance the team’s work with consequences for integration. They found that attitude implies letting yourself be limited by the expectations, if you accept recreated norms on behalf of those who are the subjects of care, and to interpret and question the standards that are imposed [28].

## Discussion

The concept of integration in Latin America has been used interchangeably with concepts such as integrated care, intersectoral approaches or integrated services to the patient, which suggests a need for further clarifications to enable outcome measures. In the included studies, it was hard to distinguish the nature of care, outcomes delivered to patients, interactions between professionals and groups, links between organizations and work groups as the clearest integration descriptors [13,14]. Often, the discussions about integrated health and care implicitly associate the systems and processes of distribution with its service: care to patient [14,38]. Here, discussion is more consistent about integration at the level of activities and mutual adjustment between health professionals, usually members of multiprofessional teams.

When studies approached relations between organizations and people, these got closer to the concept of integration as a characteristic of practices associated with work and organization. Thus, integration can be seen as something like mutual adjustment [11,19]. Here, coordination between activities and communication is seen as the main factor of integration in health. For example, a community health agent intervenes early and is forced to make decisions, which involve asking staff members for help, monitoring hospitalization, and contacting family members [21].

The interchangeable use of the concept of integration with concepts such as intersectoriality was also present in the studies. Interdisciplinarity, interprofessionality, multi/transdisciplinarity and multi/trans professionality were issues promoted among professionals. The importance of transdisciplinarity was discussed, involving not only the interactions or reciprocities between specialized knowledge and professional practices, but the placing of these relationships with patients, caregivers, community, and other agents, within an integral system free of rigid boundaries between disciplines and technical skills. From the examined papers it arose that the work of health professionals should be based on counter-referral and coordination with other specialties (administration, information technology, logistics) for the purpose of managing work processes within and between teams and organizations, directly or indirectly involved with the quality of the care in health.

In many studies, primary care was seen as a fundamental mechanism for promoting integration into the health system. PHC as a strategy to achieve integrated and universal health care systems includes five analytical dimensions: stewardship capability; funding; provision; comprehensiveness and intersectoral approach provided ways to achieve integration. In Brazil, the SUS incorporated into its guidelines the principles of Psychiatric Reform, including the process of hospitalization and guarantee of citizenship rights for those with mental illness. Such services are characterized as intermediate structures between full hospitalization and community life; they are driven by psychiatric reform projects, which have been implemented in most Brazilian states [31]. According to many studies [31,32,35,37,41,42], these kinds of actions and activities are integration mechanisms.

In two analyzed articles the principles of Suter [43] and collaborators on a successful process of integration of two health systems were mentioned. Additional principles mentioned across the articles included: comprehensive services across the care continuum; patient focus; information systems; organizational culture and leadership; physician integration; governance structure and fiscal management. The ideas that the services must attend to a continuum of care with a perspective of patient focus, cultural change, and integration of two professionals appear as recommendations in all the articles. From a managerial point of view, there is also a mention of the need to provide information systems that help in the organization of activities, alongside a governance proposal that encompasses the interested groups and promotes coordination between the units and levels of care with effective financial support.

Our results suggest that from the three main definitions of integration considered by the World Health Organisation (WHO) [44] a health system-based definition appears to be the most prevalent in Latin America. From this perspective, integration is understood as much by the process as by the result, in which result/delivery was especially emphasized in the studies analysed here. Integrated health services delivery, from this view, refers to an approach to strengthen people-centered health systems through the promotion of comprehensive delivery of quality services across the life-course. In many papers we analysed, the discourse adopted did not distinguish among the four types of integration presented by Lewis et al. [45,46] (organizational, functional, service, and clinical). Rather, the articles from Latin America assume that integration is complex and that it involves efforts from various sources and levels in the health system but provide no clarity on the typology. There is conflation between typologies, principles, mechanisms, and taxonomies that reflects the lack of a clear understanding about the phenomenon of integration as presented in the articles. The conceptual and practical links between the various levels and types of integration have not been studied in Latin America and is a major area of focus for future research.

As our analysis implies, integrated care is best understood as an emergent set of practices intrinsically shaped by contextual factors, and not as a single intervention to achieve predetermined outcomes [41]. Policies to integrate care that facilitate person-centered, relationship-based care can potentially contribute to (but not determine) improved patient experiences. There can be an association between improved patient experiences and system benefits, but these outcomes of integrated care are of different orders and do not necessarily align.

Additional studies are needed to investigate how integration processes take place at distinct levels of integration or work processes (eg., levels of integration from Rainbow Model), and in the perspective of different stakeholders and secondary information sources about people and health and care organizations. A noticeable gap in the integrated care practices described in the literature from Brazil was the absence of referral and counter-referral, which is central to SUS health policy. More experiences could be disseminated on this aspect, which is so dear to the health and care integration model. Additionally, we must address the fact that this is a narrow view of integration, so the potential for integrated care in Brazil, at least what is published, is great.

### Limitations

The aim of the article was to analyze the integration of health and care in Latin America, although most of the available articles were based in Brazil. Only two articles included experiences on health and care integration from other countries. Notable differences between experiences from Brazil and the other Latin American countries were not identified. It is important to note that the examined articles consisted of overall weak research designs, which limits the potential of drawing confident inferences from their results. Nevertheless, our analysis consolidates a hitherto dispersed body of literature upon which future research can be built, and points to gaps in the evidence base that will need addressing if integrated care is to become a reality in Latin America and other LMICs. It is surprising that few articles have been chosen on the subject considering the investments made by PAHO (Pan American Health Organization). The explanation for this may be related to the limitation of the databases or the descriptors used to detect the articles. The terms lower income, OR middle income, OR ODA eligible, OR developing countries may have restricted the pool of articles eligible for review. Due to the absence of articles involving other countries, the focus of this work was limited to Brazil.

## Conclusions

Through the examination of the available literature, the current article illustrated the key conceptual and practical factors influencing the development of, and research into integrated care in Latin America. The concept of integration presented by the studies examined reflects the different contexts, health settings and the level of attention and care studied. Consequently, a singular concept of health integration is not put forth here since contextual features need to be considered, which can be influenced by distinct patient-centered foci and institutional priorities.
